# Improving Care for Skilled Nursing Facility Residents with Opioid Use Disorder: A Qualitative Study

**DOI:** 10.1093/geroni/igaf122.1564

**Published:** 2025-12-31

**Authors:** Ashley Ritter, Corinne Roma, Charlie Merrick, Shivani Nishar, Katherine Kennedy, Jon Soske, Andrew Zullo, Patience Dow

**Affiliations:** Hunter-Bellevue School of Nursing, Hunter College, City University of New York, Yardley, Pennsylvania, United States; Brown University, Providence, Rhode Island, United States; Brown University, Providence, Rhode Island, United States; Brown University School of Public Health, Providence, Rhode Island, United States; Providence VA Healthcare System, Providence, Rhode Island, United States; Brown University Health, Providence, Rhode Island, United States; Brown University, Providence, Rhode Island, United States; Brown University School of Public Health, Providence, Rhode Island, United States

## Abstract

Skilled nursing facilities (SNFs) face financial, safety, and legal risks when transitions from hospitals are poorly managed and care practices fail to meet the needs of people with opioid use disorder (OUD). We examined strategies to optimize hospital-to-SNF transitions and access to medications for OUD (MOUD). From March to October 2023, we conducted semi-structured interviews with 29 administrative and clinical leaders involved in admissions from 27 SNFs across 19 states. Using descriptive thematic analysis, we identified five themes: (1) Variation in facility experience and stigma, and readiness: Resources and willingness to care for people with OUD varied substantially. (2) Conflation of OUD with pain management: Participants struggled to distinguish between opioids for pain, OUD, and physiologic dependence, highlighting knowledge differences about OUD. (3) Navigating information transfer: SNF staff often sought potential challenges that could negatively impact patient care before admission and noted that hospitals sometimes omitted important details to secure placement. (4) Siloed regulations and care delivery: Participants described how fragmented regulatory structures hindered admissions of people with OUD and limited their access to MOUD in SNFs. (5) Building trust and managing expectations during transition: The hospital-to-SNF transition and onboarding process was a crucial period for developing trust between people with OUD and SNF staff. Improving transitions and care for people with OUD requires education about OUD and stigma, enhanced information transfer and care coordination, and regulatory reforms to expand MOUD access in SNFs. Persistent challenges in the care for people with OUD who require SNF care require urgent action.

